# Microbial production of eumelanin from a desert-derived *Aspergillus terreus*: statistical optimization, comprehensive characterization, and in silico insights into breast cancer-associated targets

**DOI:** 10.1186/s12934-026-03114-7

**Published:** 2026-09-26

**Authors:** Shimaa El-Sapagh, El-Refaie Kenawy, Sara Ahmed, Ebrahim M. Shalamesh, Nessma A. El-Zawawy

**Affiliations:** 1 Department of Botany and Microbiology, Faculty of Science, Tanta University, Tanta, 31527 Egypt; 2 Department of Chemistry, Faculty of Science, Tanta University, Tanta, 31527 Egypt; 3 Institute for Integrative Biology of the cell, CEA, CNRS, UniversitèParis-Saclay, Gif-sur-Yvette, 91198 France

**Keywords:** Eumelanin, *Aspergillus terreus*, Optimization, Antioxidant, Breast cancer, Molecular docking

## Abstract

**Background:**

Naturally derived microbial products are increasingly explored as sustainable sources of bioactive compounds for targeted cancer applications. Among these, eumelanin is a nitrogen-containing polymeric pigment produced by various microorganisms, including fungi, and is distinguished by its unique physicochemical properties, biocompatibility, and free radical scavenging capacity. Nevertheless, eumelanin-producing fungi from extreme desert habitats remain poorly explored, particularly those isolated from the Wadi Allaqi Biosphere Reserve, and comprehensive studies integrating production optimization, structural characterization, and breast cancer-focused cytotoxic evaluation are still limited.

**Results:**

In this work, melanin was isolated from a rhizosphere soil-derived fungal isolate obtained from the Wadi Allaqi Biosphere Reserve and identified as *Aspergillus terreus* AUMC15773 through morphological examination and molecular characterization with accession number OP941720. To the best of our knowledge, this is the first report describing a melanin-producing *A. terreus* isolated from the Wadi Allaqi Biosphere Reserve, followed by comprehensive optimization, structural characterization, and breast cancer-focused cytotoxic evaluation. Melanin extraction was achieved using acid precipitation, followed by statistical optimization of culture parameters employing Plackett–Burman design and response surface methodology. This approach led to a marked increase in melanin yield, reaching 102.9 mg/10 mL compared to the initial production of 20.19 mg/10 mL indicating a 5.1 fold increase. Chromatographic analysis using thin-layer chromatography indicated a single band (Rf = 0.7), suggesting purified melanin. Structural and chemical features were confirmed using UV–visible spectroscopy, FTIR, Raman spectroscopy, and NMR, identifying the compound as DOPA-type eumelanin. Elemental profiling further supported this classification, showing a nitrogen-rich composition with low sulfur content. DPPH free radical scavenging activity assay indicated that extracted eu-melanin had antioxidant activity similar to ascorbic acid. The cytotoxic activity of the extracted eumelanin was evaluated using MCF-7 breast cancer cells and normal WI-38 fibroblasts. The purified pigment reduced the viability of MCF-7 cells while exhibiting minimal cytotoxicity toward WI-38 cells, indicating favorable biocompatibility. In silico, molecular docking revealed a stronger interaction with estrogen receptor alpha (ERα) (binding energy − 8.0 kcal/mol) compared to breast cancer susceptibility protein BRCA1 (− 6.6 kcal/mol), providing supportive computational evidence that complements the in vitro findings involving ERα modulation.

**Conclusion:**

This study establishes a desert-derived *A. terreus* isolate as a promising microbial cell factory for eumelanin production through statistical optimization and comprehensive structural characterization. The combined experimental and computational findings support the potential of the produced eumelanin for future breast cancer-related investigations. Nevertheless, further mechanistic studies and in vivo validation are required before its therapeutic applicability can be established.

**Supplementary Information:**

The online version contains supplementary material available at https://doi.org/10.1186/s12934-026-03114-7.

## Background

Breast cancer remains one of the major causes of cancer-related death among women globally, representing a substantial health burden despite advancements in detection and treatment [1–3]. Although considerable progress has been achieved in therapeutic strategies, including chemotherapy, radiotherapy, endocrine therapy, and targeted approaches [4–6], these interventions are often associated with systemic toxicity, limited specificity, and the development of drug resistance. Consequently, the development of safer and more effective bioactive compounds derived from natural sources has become increasingly important [7].

Microbial metabolites have emerged as promising candidates due to their structural diversity and potential for large-scale production. Among these, melanin is a multifunctional polymeric pigment widely distributed across different biological systems, including bacteria, fungi, plants, and animals [8–10]. Microbial production of melanin offers several advantages, including rapid growth, cost-effectiveness, scalability, and independence from seasonal and geographical limitations [11, 12]. Fungi, in particular, represent efficient platforms for melanin biosynthesis due to their metabolic versatility and adaptability to diverse environmental conditions [13]. In fungal systems, melanin is commonly associated with stress tolerance and survival under adverse conditions [14, 15], and is primarily synthesized via two major pathways: DOPA and DHN pathways [16]. Extreme environments, particularly desert ecosystems, represent valuable yet relatively underexplored reservoirs of metabolically diverse fungi with considerable biotechnological potential [17]. Nevertheless, eumelanin-producing fungi isolated from the Wadi Allaqi Biosphere Reserve have received limited attention as sustainable microbial sources of eumelanin.

Optimizing melanin production is essential to enhance its applicability. Conventional approaches such as the one-factor-at-a-time method are often inefficient, time-consuming, and fail to account for interaction effects between variables [18, 19]. In contrast, statistical optimization techniques such as Plackett–Burman design [20] and response surface methodology [21] enable efficient screening and optimization of multiple factors simultaneously, leading to improved production yields. Melanin has been reported to exhibit a broad spectrum of physicochemical and biofunctional properties, including free radical scavenging activity and cytotoxic effects against selected cancer cell lines [22, 23]. However, its interaction with key molecular targets involved in breast cancer progression, such as estrogen receptor alpha (ERα) and BRCA1, remains poorly understood. Computational approaches, particularly molecular docking, have become valuable tools for predicting molecular interactions and generating mechanistic hypotheses during early-stage drug discovery processes [24].

Accordingly, the present study aimed to isolate and identify a melanin-producing fungal strain from rhizosphere soil collected from the Wadi Allaqi Biosphere Reserve, optimize melanin production using statistical design approaches, characterize the extracted melanin through comprehensive physicochemical analyses, and evaluate its relevance to breast cancer through targeted in vitro cytotoxicity assessment and in silico analysis focusing on ERα and BRCA1.

## Methodology

### Soil sampling, fungal isolation and purification

Rhizosphere soil samples (*n* = 8) were collected from different locations within the Wadi Allaqi Biosphere Reserve, located in the southeastern desert of Egypt, approximately 180 km south of Aswan along the eastern shore of Lake Nasser, during May 2022. Samples were aseptically transferred into sterile polyethylene bags, homogenized, and transported under cooled conditions to the Mycology Laboratory, Department of Botany and Microbiology, Faculty of Science, Tanta University, Egypt. Upon arrival, samples were air-dried and processed for fungal isolation following standard procedures [25].

Fungal isolation was performed using a serial dilution and spread plate technique as depicted by Celestino et al. [26]. Briefly, 10 g of each soil sample was suspended in 90 mL of sterile distilled water, thoroughly mixed, and serially diluted up to 10⁻⁵. Separately, 0.1 mL of each dilution was added to potato dextrose agar (PDA; HiMedia, Mumbai, India; Ref. GM075) that had been treated with chloramphenicol (100 mg/L) in order to minimize bacterial growth. Plates were incubated at 28 ± 2 °C for 7–10 days. Each fungal isolate was subcultured onto fresh PDA plates to obtain pure isolates. Purification was achieved using repeated subculturing techniques [27]. The purified isolates were preserved in 70% glycerol and stored at − 20 °C for further analysis.

### Screening of melanin production

A total of 21 fungal isolates were preliminarily evaluated for their ability to produce melanin using modified potato dextrose broth (PDB) supplemented with l-tyrosine 5 g/L [28]. Each isolate was inoculated onto sterile media and incubated for 7 days under controlled conditions. The appearance of a brown to dark-colored diffusible pigment in the surrounding medium was considered indicative of melanin production. Among the tested isolates, one strain exhibited pigment production and was designated as F11. This isolate was selected for further molecular identification and subsequent investigations.

### Morphological and molecular identification

Morphological characterization of the selected fungal isolate (F11) was carried out according to standard taxonomic criteria described by Samson et al. [29]. The isolate was cultured on PDA plates, and colony characteristics including growth rate, texture, and pigmentation of both aerial and substrate mycelia were recorded after 7 days of incubation. For molecular identification, the isolate was cultivated in potato dextrose broth (PDB) at 28 °C with agitation at 150 rpm for 7 days. The Molecular Biology Research Unit, Assiut University, collected genomic DNA from freshly grown mycelia using the Patho-gene-spin DNA/RNA extraction kit (Intron Biotechnology, Korea) according to the manufacturer’s procedure. ITS1 and ITS4 universal primers amplified the rRNA gene’s internal transcribed spacer (ITS) region [30]. PCR products were purified and sequenced using the same primer pair (SolGent Co., Daejeon, South Korea). The closest phylogenetic relationship was determined by comparing the acquired sequences to reference sequences in the NCBI database using the BLAST program. The validated sequence was submitted to GenBank to obtain an accession number. Multiple sequence alignment was performed, and a phylogenetic tree was constructed using the neighbor-joining method implemented in MegAlign (DNASTAR, version 5.05) [31].

### Optimization of nutritional and physical parameters for melanin production

To evaluate the influence of cultural and nutritional variables on melanin production, a Plackett–Burman design (PBD) was applied using Design-Expert software (Stat-Ease Inc., Minneapolis, USA) [20]. A Plackett–Burman design was used to screen the effects of 11 independent nutritional and physical variables (pH, temperature, inoculum size, incubation time, tyrosine, potato extract, dextrose, sodium dihydrogen phosphate, copper sulfate, ferrous sulfate, and calcium chloride) on melanin production. The experimental matrix consisted of 12 runs, with each factor evaluated at two coded levels, − 1 and + 1, as summarized in Table S1. The selected ranges of variables were based on previously reported studies [32, 33]. No dummy variables were included in the Plackett–Burman design. The statistical reliability of the identified significant factors was evaluated based on analysis of variance (ANOVA), model F-value, p-value, coefficient of determination (R²), adjusted and predicted R² values, coefficient of variation (CV), Adeq Precision, and subsequent experimental validation under the optimized conditions [34]. The Plackett–Burman design follows a first-order linear model:

1 $${\mathbf{Y}}=\boldsymbol{\upbeta}{\mathbf{0}}+\sum \boldsymbol{\upbeta}{\mathbf{i}}\;{\mathbf{Xi}}$$

where *Y* represents melanin production, *β₀* is the intercept, *βi* denotes the linear coefficients, and *Xi* represents the coded independent variables [35, 36]. Since the Plackett–Burman design is a first-order screening design, interaction effects among variables were assumed to be negligible and were therefore not included in the statistical model. The primary objective of this design was to identify the most influential factors affecting melanin production for subsequent optimization by Box–Behnken Design.

Based on the PBD results, the three most influential factors were selected for further optimization using response surface methodology (RSM) with a Box–Behnken design (BBD) [37]. The design matrix consisted of 17 experimental runs, including five replicates at the center point to estimate experimental error and ensure model reliability (Table S2). Each variable was evaluated at three levels (− 1, 0, + 1), corresponding to low, intermediate, and high values (Table 2). A second-order polynomial equation was developed to describe the relationship between the independent variables and melanin production:

2 $${\mathbf{Y}}=\boldsymbol{\upbeta}{\mathbf{0}}+\sum \boldsymbol{\upbeta}{\mathbf{i}}\;{\mathbf{Xi}}+\sum \boldsymbol{\upbeta}{\mathbf{ij}}\;{\mathbf{Xi}}\;{\mathbf{X}}\;{\mathbf{j}}+\sum \boldsymbol{\upbeta}{\mathbf{ii}}{\text{ }}{\mathbf{X}}{{\mathbf{i}}^{\mathbf{2}}}$$

where *Y* is the predicted response, *β₀* is the intercept term, *βi* represents linear coefficients, *βiⱼ* corresponds to interaction effects, and *βii* denotes quadratic coefficients [38].

Three-dimensional response surface plots were generated to visualize the individual and interactive effects of significant variables and to determine their optimal levels for maximum melanin production by *A. terreus*.

### Extraction and quantification of melanin pigment

Melanin produced by the selected isolate F11 under submerged fermentation was extracted according to acid precipitation method [39]. Briefly, the fungal culture was incubated for 14 days under optimized conditions to promote extracellular pigment production. The culture broth was then acidified to pH 2.0 using 6 M HCl and maintained at room temperature for 24 h to allow pigment precipitation. The resulting precipitate was collected by centrifugation at 4500×*g* for 15 min, followed by repeated washing with distilled water to remove residual impurities. The purified pigment was subsequently freeze-dried to obtain a dry melanin powder [40]. For quantitative determination, the dried pigment was dissolved in 0.5 M NaOH to a final concentration of 1 mg/mL. Absorbance was measured at 280 nm using a UV–visible spectrophotometer. Quantification was achieved using a standard calibration curve prepared from synthetic melanin (Sigma-Aldrich, USA; Ref. M8631) [41].

### Purification of melanin pigment

The purity of the extracted melanin was assessed using thin-layer chromatography (TLC) [42]. Both the purified melanin extract and the reference melanin standard were dissolved in dimethyl sulfoxide (DMSO) at a concentration of 1 mg/mL to ensure complete solubilization. Aliquots (5 µL) of each solution were applied onto silica gel plates (Merck TLC Silica Gel 60 F254) serving as the stationary phase. Chromatographic development was carried out using a mobile phase composed of petroleum ether, ethyl acetate, 95% ethanol, and ammonia (4:4:6:1, v/v). Following solvent migration, the plates were air-dried and examined under ultraviolet light. The retention factor (R_f_) of the developed pigment was determined and compared to that of conventional melanin to confirm sample purity.

### Physiochemical and spectroscopic characterizations of the extracted melanin

The physicochemical properties of the extracted melanin obtained from selected isolate F11 were evaluated following reported procedures by Rudrappa et al. [43]. The pigment’s solubility was examined at a concentration of 5 mg/mL in a range of organic and inorganic solvents, including distilled water, 1M NaOH, 1 N HCl, dimethyl sulfoxide (DMSO), methanol, absolute ethanol, chloroform, benzene, acetone, and ethyl acetate. Samples were vortexed for 10 min at 7000 rpm and incubated for 2 h, after which solubility was visually assessed. Chemical characterization included ferric chloride (FeCl₃) testing to confirm the presence of phenolic groups and precipitation in 3 M HCl to verify melanin characteristics. Oxidative stability was evaluated by treating melanin solutions (0.1 g/L) with hydrogen peroxide (30%) and potassium dichromate (K₂Cr₂O₇) [44]. Thermal stability was assessed by exposing the pigment to 60, 80, and 100 °C for 2, 4, and 6 h. All results were compared with those obtained from standard melanin.

UV–visible spectroscopic analysis was performed using a UV-2600 spectrophotometer (Shimadzu, Japan). The melanin sample (1 mg/10 mL in 1 N NaOH) was scanned over a wavelength range of 200–700 nm, using 1 N NaOH as a blank, and the maximum absorption wavelength (λ_max_) was recorded [45]. Fourier transform infrared (FTIR) spectroscopy was performed to detect the functional groups present in the pigment. The sample was prepared by mixing melanin with IR-grade potassium bromide (KBr) at a ratio of 1:10, followed by pellet formation under vacuum. Spectra were recorded using a PerkinElmer FTIR spectrometer (USA) within the range of 400–4000 cm⁻¹ at a resolution of 4 cm⁻¹ [46]. Data were processed using the corresponding spectral analysis software. UV–Vis and FTIR analyses were carried out at the Scientific Research Center and Measurement (SRCM), Tanta University, Egypt.

For Raman analysis, equal volumes of melanin suspension and double-distilled water were mixed and sonicated for 20 min. A drop of the mixture was placed onto a glass slide and allowed to dry at 25 ± 2 °C for 48 h. The sample was then analyzed using a Bruker RFS 27 spectrometer. FT-Raman spectra were recorded using SENTERRA II Raman microscopy (Bruker) to identify vibrational modes [47]. Nuclear magnetic resonance (¹H and ¹³C NMR) analysis was performed by dissolving 20 mg of extracted melanin in 1 mL of DMSO. The solution was filtered and transferred into a 5 mm NMR tube. Spectra were recorded using a JEOL GSX 400 spectrometer at a frequency of 400 MHz and an acquisition time of 1.63 s. Data were analyzed using DELTA 5.3.1 software [48]. This analysis was conducted at the National Research Center, Giza, Egypt. Elemental composition was determined using energy-dispersive X-ray spectroscopy (EDX) [49], while surface morphology was examined using scanning electron microscopy (SEM) (JEOL JSM-5200 LV, Japan) operated at an accelerating voltage of 20 kV. SEM, EDX, and Raman analyses were performed at Nano Gate (Cairo, Egypt).

### DPPH free radical scavenging assay

The free radical-scavenging activity of extracted melanin pigment from *Aspergillus terreus* AUMC15773 strain was assessed using 2, 2-diphenyl-1 picrylhydrazyl (DPPH) assay [50]. In brief, a standard stock solution was prepared by dissolving 4 mg of 0.02 mM DPPH in 100 ml of methanol and then storing the mixture at 4 °C. A mixture of two milliliters of freshly prepared stock solution and one milliliter of extracted melanin pigment were mixed at different concentrations (1.25, 2.5, 5, 10, 25, 50 and 100 µg/mL). Methanol served as the experiment’s negative control, while ascorbic acid (Vitamin C) served as the positive control. At room temperature, this combination was allowed to stand for half an hour in the dark. At 517 nm, the absorbance was measured using JENWAY 6305 ultraviolet-visible spectrophotometer. The free-radical scavenging activity (%) was calculated using the formula:

3 $$\begin{aligned} {\mathbf{Scavenging}}\;{\mathbf{activity}}\left( \% \right) & =\left( {{\mathbf{Absorbance}}\;{\mathbf{at}}\;{\mathbf{blank}}} \right) - \left( {{\mathbf{Absorbance}}\;{\mathbf{at}}\;{\mathbf{test}}} \right) \\ & \quad /\left( {{\mathbf{Absorbance}}\;{\mathbf{at}}\;{\mathbf{blank}}} \right)] \times {\mathbf{100}} \\ \end{aligned} $$

The concentration of melanin required to scavenge 50% of the radicals (IC_50_) was determined using a linear regression curve.

### In vitro cytotoxicity assay

The cytotoxic effect of the extracted melanin from selected isolate F11 was evaluated on both normal and cancer cell lines. The normal human lung fibroblast cell line (WI-38) and breast adenocarcinoma (MCF-7) were obtained from the American Type Culture Collection (ATCC) through VACSERA (Cairo, Egypt). Cell viability was assessed using the MTT assay according to Chen et al. [51]. A density of 1 × 10⁴ cells per well was used to seed cells into 96-well plates, which were then incubated at 37 °C in a humidified environment containing 5% CO₂ over 48 h. Following incubation, cells were treated with 100 µL per well of serially diluted melanin at concentrations of 0.05, 0.5, 5, 50, and 500 µg/mL. After 24 h of treatment, the culture medium was removed, and 40 µL of MTT solution (5 mg/mL) was added to each well, followed by incubation for an additional 4 h. Dimethyl sulfoxide (DMSO) was added to dissolve the formazan crystals, and a microplate reader (EXL 800, USA) was used to detect absorbance at 570 nm.

Cell viability (%) was calculated using the following equation:

4 $${\mathbf{CV}}\% =\left( {{\mathbf{Absorbance}}\;{\mathbf{of}}\;{\mathbf{treated}}\;{\mathbf{samples}}/{\mathbf{Absorbance}}\;{\mathbf{of}}\;{\mathbf{untreated}}\;{\mathbf{sample}}} \right) \times {\mathbf{100}}$$

The half-maximal inhibitory concentration (IC₅₀) values were calculated by nonlinear regression analysis using GraphPad InStat software (version 3.1). All experiments were performed in triplicate, and the results are presented as mean ± standard deviation (SD).

### Molecular docking analysis

In silico molecular docking was performed to evaluate the potential interactions between the extracted melanin and key proteins implicated in breast cancer. The three-dimensional (3D) structures of breast cancer type 1 susceptibility protein (BRCA1; PDB ID: 1JNX) and estrogen receptor alpha (ERα; PDB ID: 3ERT) were retrieved from the RCSB Protein Data Bank. The 3D structure of the ligand was obtained from the PubChem database (CID: 102582077) and subjected to energy minimization using Avogadro software (version 1.2.0). Protein preparation was carried out using AutoDockTools (version 1.5.7) [52], which included removal of water molecules and non-essential heteroatoms, addition of polar hydrogen atoms, assignment of Kollman charges, and correction of missing structural elements. The prepared protein structures were saved in pdbqt format for docking analysis. The active sites of the selected proteins were identified, and grid boxes were defined to encompass the binding regions, with a grid spacing of 0.375 Å. Molecular docking simulations were conducted using AutoDock Vina [53] to estimate binding affinities, interaction energies, and potential hydrogen bonding patterns between the ligand and target proteins. Additionally, both two-dimensional (2D) and three-dimensional (3D) interaction profiles were generated to visualize the binding modes and key interactions between the ligand and the target proteins. Docking outputs were further analyzed and visualized using BIOVIA Discovery Studio software (version 21.1.0.20298).

## Results and discussion

### Isolation and screening of fungi for melanin production

Rhizosphere soil is recognized as a rich ecological niche harboring diverse microbial communities, particularly fungi, which are renowned for their capacity to generate a diverse array of bioactive metabolites. Among these, melanin represents a structurally diverse pigment with significant biotechnological potential, and fungal systems are considered efficient platforms for its large-scale production [54]. In the present study, eight rhizosphere soil samples were collected from different sites within the Wadi Allaqi Biosphere Reserve, a unique desert ecosystem characterized by harsh environmental conditions, including intense solar radiation, temperature fluctuations, and limited water availability. Such stressors are known to stimulate the production of protective secondary metabolites in fungi, including melanin, which plays a crucial role in enhancing tolerance to oxidative stress, desiccation, and ultraviolet exposure [55]. Therefore, this extreme habitat may represent a selective ecological reservoir for stress-adapted, melanin-producing fungi with potential value for microbial pigment production.

A total of 21 morphologically distinct fungal isolates were recovered from the collected samples using the serial dilution method on PDA medium. Preliminary screening for melanin production revealed that only one isolate (F11) exhibited a pronounced ability to synthesize a diffusible dark brown pigment on an L-tyrosine-supplemented medium (Fig. S1). The selection of a single positive isolate among the screened fungal collection highlights the restricted distribution of this phenotype under the tested cultivation conditions. The observed pigmentation is likely associated with the enzymatic oxidation of L-tyrosine by tyrosinase, leading to the formation of intermediate compounds such as L-DOPA and subsequent polymerization into melanin [56]. The initiation of melanin synthesis requires the incorporation of different precursors, with L-tyrosine serving as a cost-effective substrate for industrial melanin production [57, 58]. Moreover, Ben-Tahar et al. [59] documented that the incorporation of L-tyrosine markedly enhanced melanin synthesis in *A. auricula* and the yeast *Y. lipolytica* W29.

### Morphological and molecular characterization of F-11 isolate

**Fig. 1:**
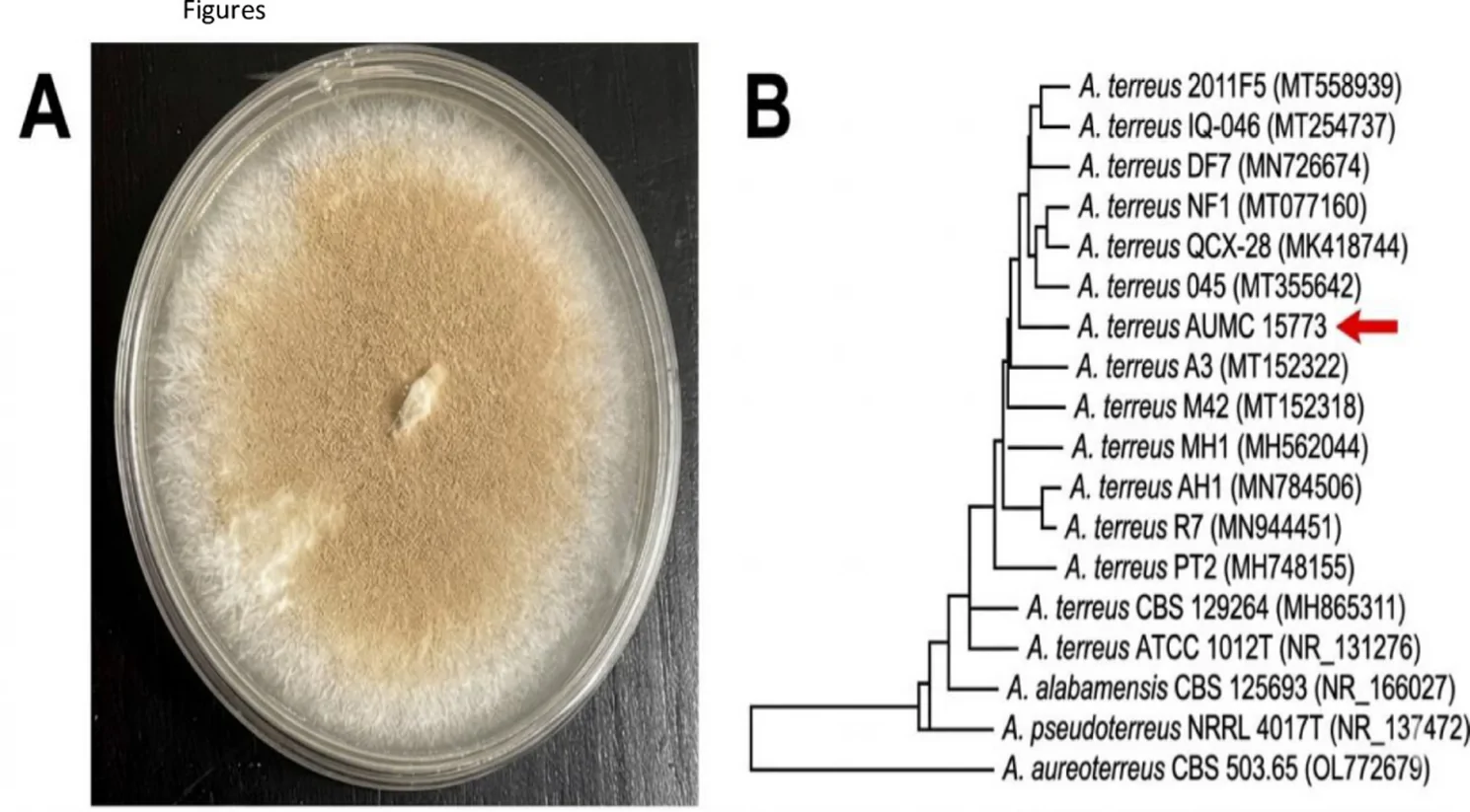
Morphological characters of *Aspergillus terreus* AUMC15773 strain (**A**). Phylogenetic tree based on ITS sequences of rDNA (*Aspergillus terreus* AUMC15773 strain with Gene Bank accession no. OP941720, arrowed) aligned with closely related sequences of strains accessed from the fungal GenBank (**B**)

The selected fungal isolate (F11) was identified based on a combination of morphological, and molecular characteristics. Macroscopically, the isolate formed large, irregular, wrinkled colonies with a buff to beige coloration on PDA (Fig. 1A). For molecular identification, the internal transcribed spacer (ITS) region was amplified and sequenced. The obtained sequence was 580 base pairs in length and was analyzed using the BLAST search computational software against the NCBI database. The sequence was deposited in GenBank under the accession number OP941720. Phylogenetic analysis based on the neighbor-joining method (Fig. 1B) demonstrated that the isolate clustered closely with reference strains of *Aspergillus terreus*, showing 100% sequence identity and full query coverage, including the type strain *A. terreus* ATCC 1012T (NR_131276). Based on the combined morphological and molecular evidence, the isolate was identified as *Aspergillus terreus* and designated as *A. terreus* AUMC15773. These findings are in agreement with previous reports indicating that *Aspergillus* species are capable of producing various forms of melanin, which contribute to their adaptability and survival under environmental stress conditions [60]. To the best of our knowledge, this is the first study to investigate melanin production by an *A. terreus* isolate recovered from the Wadi Allaqi Biosphere Reserve using an integrated optimization and characterization strategy.

### Melanin quantification and optimization

**Fig. 2:**
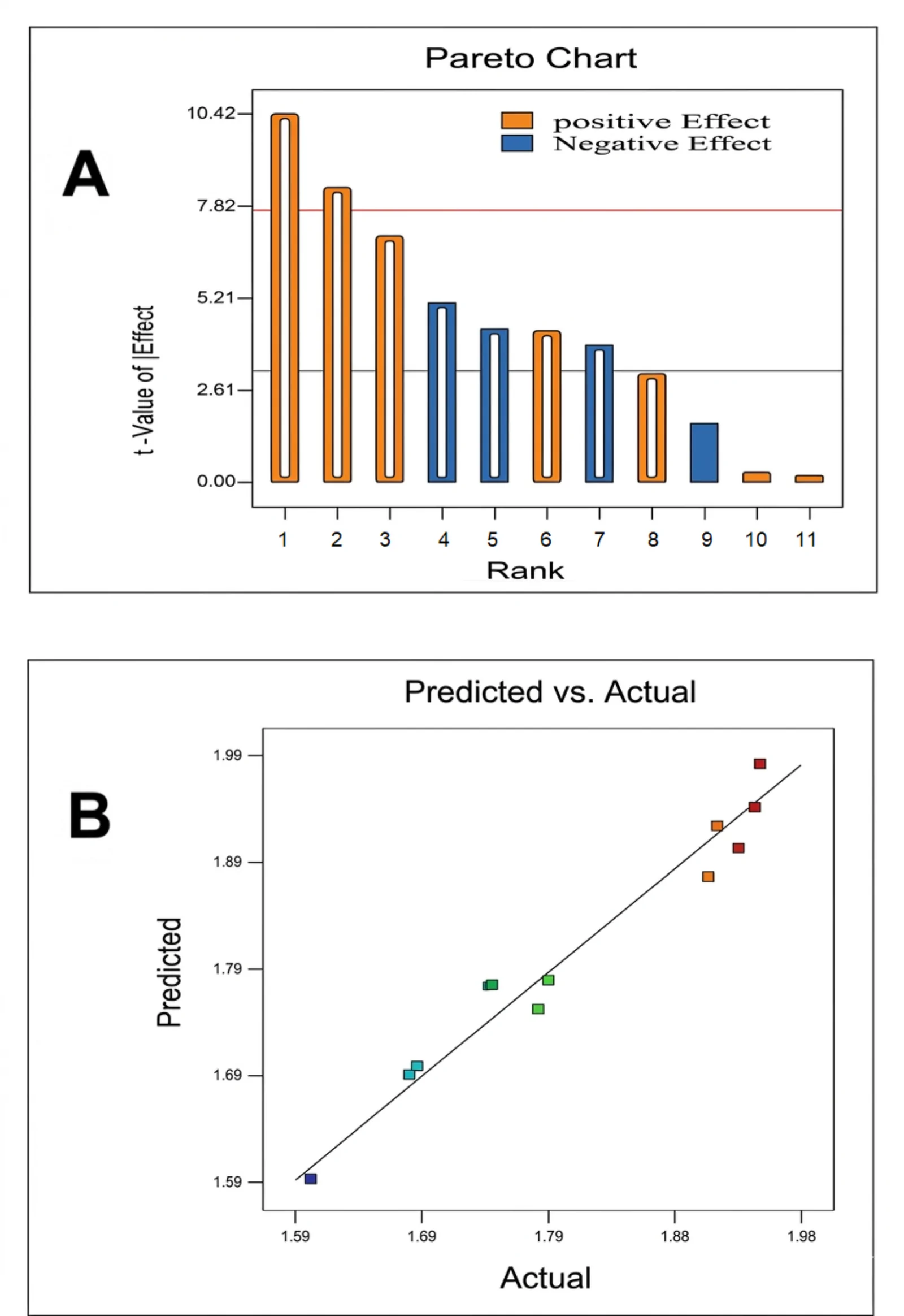
Pareto chart depicts the degree to which each variable influences production of melanin (**A**); Correlation between the experimentally actual and predicted values for melanin production by *A. terreus* according to the Plackett–Burman experimental results (**B**)

Melanin production by *A. terreus* was initially quantified spectrophotometrically, yielding 20.19 mg/10 mL after 7 days of cultivation in modified PDB supplemented with L-tyrosine. This variation is consistent with previous reports demonstrating that melanin biosynthesis is strongly influenced by medium composition and environmental conditions [61]. To identify the key variables affecting pigment production, a Plackett–Burman design (PBD) was employed. The experimental results in Table 1 revealed a marked variation in melanin yield across different trials, ranging from 40.28 to 89.16 mg/10 mL indicating the significant influence of the tested factors as in Table 2. These findings are similar with previous studies emphasizing the role of nutritional and environmental parameters in fungal pigment biosynthesis [13, 62]. Moreover, the Pareto chart illustrated the relative significance of the investigated variables, highlighting that L-tyrosine was identified as the most influential factor, followed by sodium dihydrogen phosphate and inoculum size as in Fig. 2A. The predicted versus actual plot further confirmed the adequacy of the model, demonstrating close agreement between experimental and predicted values (Fig. 2B). The prominent role of tyrosine can be attributed to its function as a key precursor in melanin biosynthesis pathways [63, 64], while phosphate sources are known to enhance metabolic activity and pigment production [65]. In addition, inoculum size significantly affects biomass formation and metabolic output, as previously reported [66].

**Table 1:** Plackett-Burman experimental design for evaluation of twelve independent variables with codes for myco-synthesis of melanin by *A. terreus* strain as a response reflecting the yield

Run No.	A: pH	B: Temperature	C: Inoculum volume	D: Incubation time	E: Tyrosine	F: Potato	G: Ferrous sulphate	H: Sodium dihydrogen phosphate	J: Dextrose	K: Copper Sulphate	L: Calcium chloride	Production of melanin (mg/10 mL)
1	9	35	0.5	14	7	250	1.5	10	1	1	0.5	55.343
2	5	35	2.5	7	7	250	2.5	10	1	0.1	1	60.164
3	9	25	2.5	14	3	250	2.5	14	1	0.1	0.5	85.86
4	5	35	0.5	14	7	150	2.5	14	3	0.1	0.5	81.353
5	5	25	2.5	7	7	250	1.5	14	3	1	0.5	89.161
6	5	25	0.5	14	3	250	2.5	10	3	1	1	40.28
7	9	25	0.5	7	7	150	2.5	14	1	1	1	88.434
8	9	35	0.5	7	3	250	1.5	14	3	0.1	1	47.92
9	9	35	2.5	7	3	150	2.5	10	3	1	0.5	61.328
10	5	35	2.5	14	3	150	1.5	14	1	1	1	55.568
11	9	25	2.5	14	7	150	1.5	10	3	0.1	1	82.763
12	5	25	0.5	7	3	150	1.5	10	1	0.1	0.5	48.608

**Table 2:** Regression statistics and analysis of variance (ANOVA) for the experimental results of Plackett Burman design of melanin myco- production by *A. terreus*

Source	SS	Df	MS	F-value	*P*-value
Model	3509.05	9	389.89	86.04	0.0115*
A-pH	180.3	1	180.3	39.79	0.0242*
B-Temperature	449.33	1	449.33	99.16	0.0099*
C-Inoculum volume	442.94	1	442.94	97.74	0.0101*
E- L-tyrosine	1153.54	1	1153.54	254.55	0.0039*
F-Potato	128.88	1	128.88	28.44	0.0334*
G- Ferrous sulphate	120.69	1	120.69	26.63	0.0356*
H-Sodium dihydrogen Phosphate	830.17	1	830.17	183.2	0.0054*
K-Copper sulphate	22.84	1	22.84	5.04	0.1539
L-Calcium chloride	180.37	1	180.37	39.8	0.0242*
Residual	9.06	2	4.53		
Cor Total	3518.11	11			
Std. Dev.	2.13		R- Squared	0.9974	
Mean	66.4		Adjusted R-Squared	0.9858	
C.V. %	3.21		Predicted R-Squared	0.9073	
PRESS	326.27		Adeq Precision	26.013	

1

**Fig. 3:**
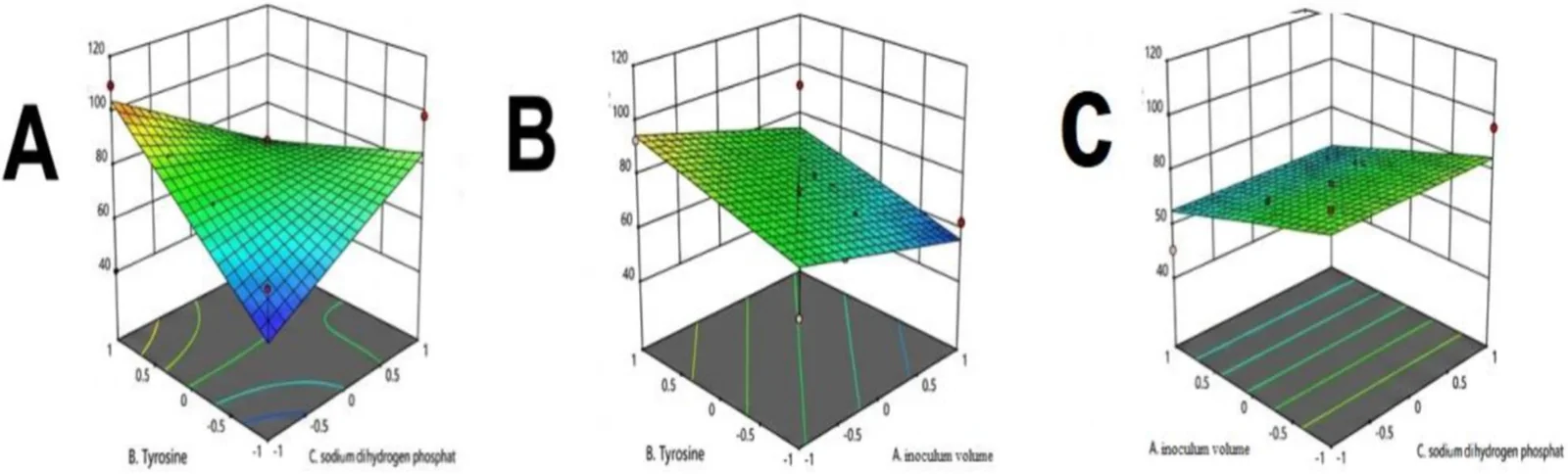
The 3D surface response showing the effect of inoculum volume (**A**); l-tyrosine (**B**); and sodium dihydrogen phosphate (**C**) and their mutual interaction on melanin production by *A. terreus*

**Fig. 4:**
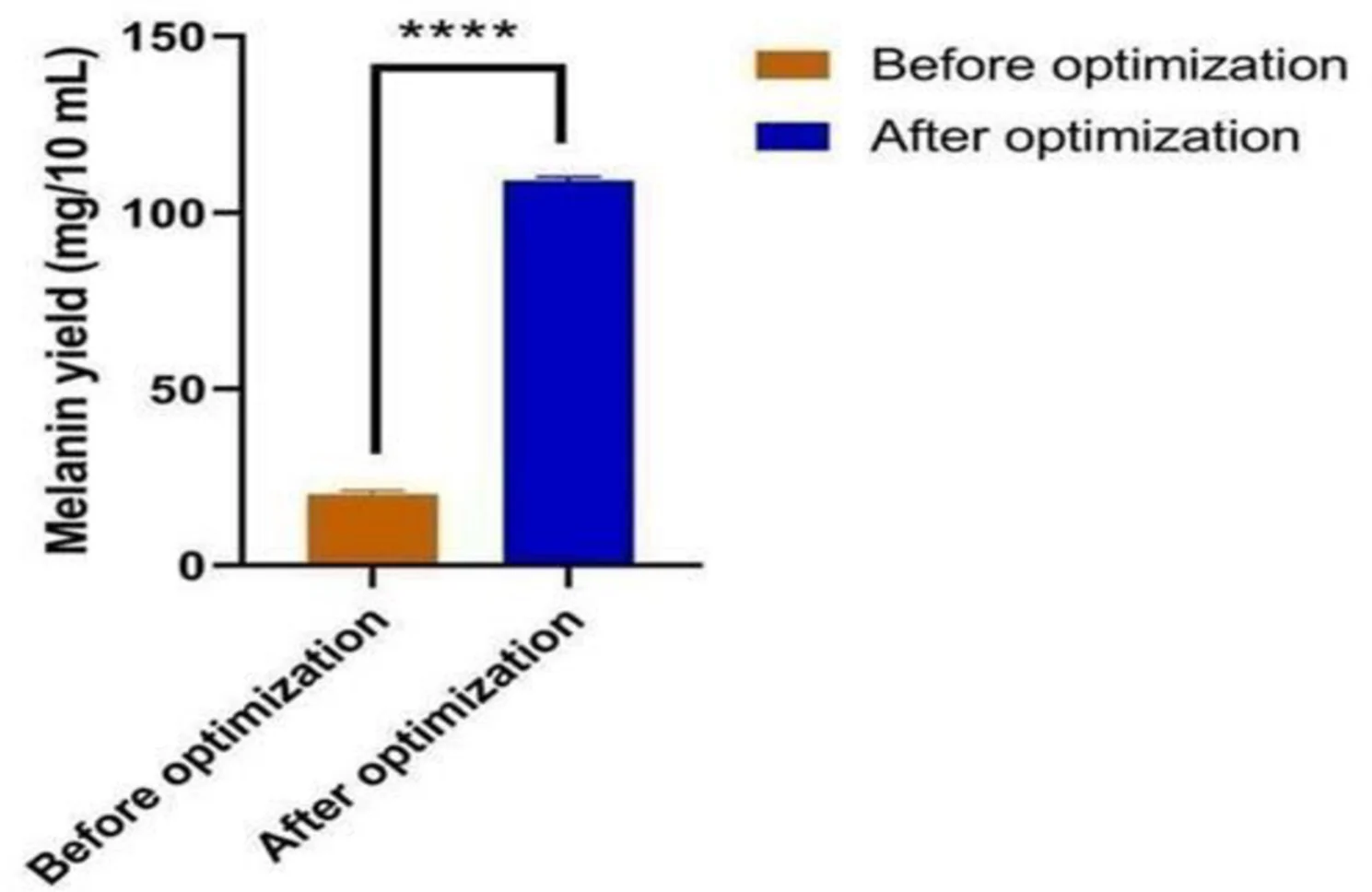
Melanin production by *A. terreus* before and after RSM optimization

Based on the PBD results, the three most significant variables (inoculum size, tyrosine, and sodium dihydrogen phosphate) were further optimized using Box–Behnken design (BBD). The experimental matrix demonstrated variation in melanin yield, with the highest response observed in run 7 (Table 3). ANOVA results in Table 4 confirmed the statistical significance of the model (F = 4.31, *p* = 0.0217), and an interaction was observed between tyrosine and sodium dihydrogen phosphate, whereas other variable combinations showed comparatively minor effects on melanin production. In order to identify the variables’ interactions and the ideal values for each variable required for maximum production, three-dimensional response surface curves were generated. Figure 3 displays the 3D response surface plots for important combinations of the three variables in pairs. With the third variable held constant at zero (intermediate value), the melanin yield was plotted on the z-axis of each three-dimensional response surface plot (Fig. 3A, B, and C) against two independent variables that were explored simultaneously. The two variables, B and C, that were investigated interacted significantly. The correlation between other variables doesn’t help much in increasing the yield. BBD optimization predicted a maximum yield of 109.2 mg/10 mL under optimal conditions representing a 5.1-fold increase compared to the initial production level as in (Fig. 4). This significant improvement highlights the efficiency of statistical optimization strategies in enhancing microbial melanin production. Similar trends have been reported in other microbial systems using comparable approaches [67].

**Table 3:** Seventeen trials of Box-Behnken design representing melanin production by *A. terreus*

Run No.	Experimental parameters			Melanin yield (mg/10 mL)	
	A	B	C	Actual value	Predicted value
1	− 1	− 1	0	70.50	75.21
2	1	− 1	0	55.50	64.98
3	− 1	1	0	62.50	55.98
4	1	1	0	50.30	65.50
5	− 1	0	− 1	70.20	75.21
6	1	0	− 1	57.92	75.21
7	− 1	0	1	109.20	109.639
8	1	0	1	92.50	85.45
9	0	− 1	− 1	95.80	84.93
10	0	1	− 1	98.50	84.89
11	0	− 1	1	71.50	75.21
12	0	1	1	58.50	75.93
13	0	0	0	65.40	47.01
14	0	0	0	91.50	74.50
15	0	0	0	70.50	75.21
16	0	0	0	92.80	94.45
17	− 1	0	− 1	65.50	65.01

**Table 4:** Regression statistics and ANOVA for the experimental results of BBD results

Source	SS	Df	MS	F-value	*p*-value	
Model	2957.47	4	739.37	4.31	0.0217*	Significant
A-Inoculum volume	796.00	1	796.00	4.64	0.0523	
B- l-tyrosine	686.35	1	686.35	4.00	0.0687	
C-Sodium dihydrogen phosphate	0.5513	1	0.5513	0.0032	0.9557	
BC	1474.56	1	1474.56	8.59	0.0126	
Lack of Fit	1929.28	8	241.16	7.36	0.0356*	Significant
Residual	2060.40	12	171.70	Model R-Squared		0.5894
Pure Error	131.12	4	32.78	Adjusted R- Squared		0.4525
Cor Total	5017.87	16		Predicted R- Squared		− 0.1205

*Significant values, SS- sum of squares, MS- mean of square, F: Fishers, s function, P: Level of significance, CV %- the coefficient of variation %

### Extraction and purification of melanin

Melanin extraction approaches vary depending on the producing organism, the intracellular or extracellular localization of the pigment, and its physicochemical characteristics. In fungal systems, melanin may either be associated with cellular structures or secreted into the surrounding medium [32]. Extracellular melanin is commonly obtained through acid precipitation following solubilization under alkaline conditions or direct acidification [68]. In the present study, the extracellular melanin pigment produced by *A. terreus* was isolated from the culture broth via acid precipitation using 6M HCl, resulting in the formation of a dark insoluble precipitate. Following extraction, the purity of the pigment was assessed using thin-layer chromatography (TLC). The analysis was performed on silica gel plates using an optimized solvent system consisting of petroleum ether, ethyl acetate, 95% ethanol, and ammonia (4:4:6:1, v/v). After chromatographic development, the plates were examined under ultraviolet illumination (280 nm), where the melanin pigment appeared as a distinct band due to its conjugated aromatic structure, which exhibits strong UV absorption. The retention factor (R_f_) value of the extracted melanin pigment was calculated to be 0.7, showing a single well-resolved band comparable to that of the reference melanin (Fig. S2), indicating a high degree of purity. Similar observations have been reported for melanin extracted from *Penicillium citrinum*, which exhibited a single band with a comparable R_f_ value (0.68) under similar conditions [43].

### Characterization of the extracted melanin

#### Physiochemical properties of extracted melanin pigment

Preliminary identification of extracted melanin is commonly based on its characteristic physicochemical behavior. Accordingly, the solubility profile of the extracted pigment was evaluated in a range of organic and inorganic solvents (Table S3). The extracted dark-brown pigment exhibited good solubility in water and organic solvents such as ethanol, methanol, chloroform, acetone, benzene, and ethyl acetate, while enhanced solubility was observed in dimethyl sulfoxide (DMSO) and alkaline solutions, including sodium hydroxide and potassium hydroxide. These findings indicated the typical solubility behavior of melanin, which is known to dissolve readily under alkaline conditions. Thermal stability analysis demonstrated that the pigment maintained its integrity over a wide temperature range, indicating a high degree of thermostability. In addition, the pigment readily precipitated upon acidification (3N HCl), further confirming its characteristic pH-dependent solubility. Chemical reactivity assays showed that the pigment produced a brown flocculent precipitate upon treatment with ferric chloride (FeCl₃), confirming the presence of phenolic functional groups. Furthermore, the pigment underwent decolorization when exposed to oxidizing agents such as hydrogen peroxide (30%, v/v) and potassium permanganate, indicating its susceptibility to oxidative degradation. Overall, the observed physicochemical properties are consistent with previously reported characteristics of melanin from various microbial sources [69–71]. Similar solubility and reactivity patterns have been described for extracellular melanin produced by *Nocardiopsis* spp. [46]. and *Auricularia auricular* [72], which demonstrated solubility in alkaline solutions and precipitation under acidic conditions, along with sensitivity to oxidative agents.

#### Spectroscopic analysis of extracted melanin pigment

**Fig. 5:**
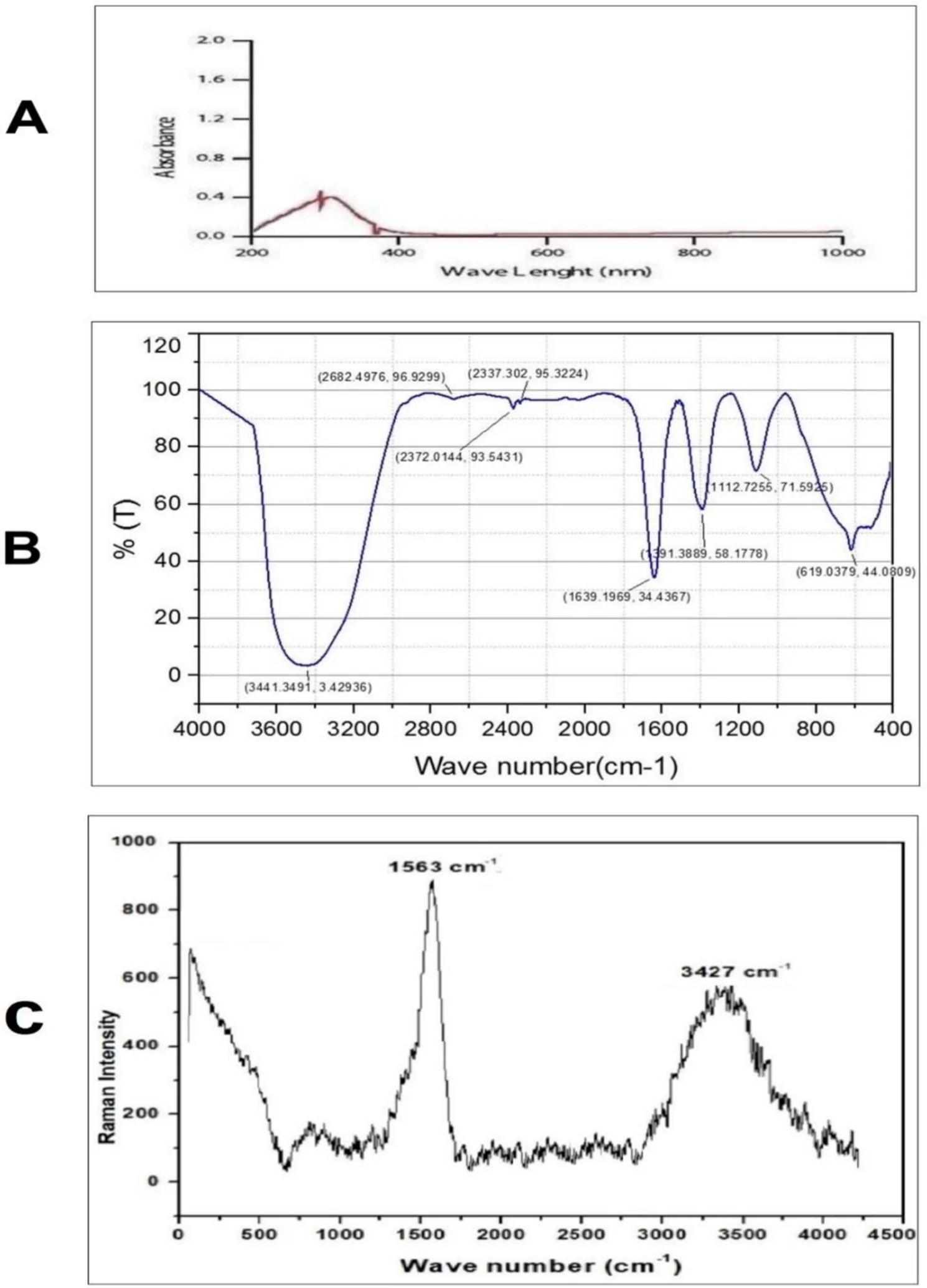
UV absorption spectra (**A**), FTIR (**B**), and Raman spectroscopy (**C**) of extracted melanin produced by *A. terreus*

The extracted melanin obtained from *A. terreus* was subjected to spectroscopic analyses to confirm its identity and elucidate its structural features. UV–visible spectroscopy revealed a characteristic absorption profile, with a strong peak observed at 225 nm within the ultraviolet region (Fig. 5A) and a gradual decline in absorbance toward the visible range. This pattern is consistent with the typical behavior of melanin, which exhibits broad UV absorption due to its highly conjugated aromatic structure [9, 73, 74]. The absence of distinct peaks in the visible region further supports the identification of the pigment as melanin. Similar absorption profiles have been reported for microbial melanins, which generally display maximum absorbance within the 200–400 nm range [75]. Comparable findings were also observed for melanin produced by *Phoma* sp., which showed a peak in the UV region around 230 nm [76].

To further investigate the functional groups present in the pigment, Fourier transform infrared (FTIR) spectroscopy was performed (Fig. 5B). The spectrum displayed a broad band at 3441 cm⁻¹ corresponding to O–H stretching vibrations, along with peaks at 2337 cm⁻¹ (CH₂ stretching) and 1639 cm⁻¹, which can be attributed to C=O or aromatic C=C stretching [77, 78]. These features suggest the presence of conjugated carbonyl groups associated with aromatic ring structures. Additional bands at 1391 cm⁻¹ (C–N stretching) indicate the presence of nitrogen-containing functional groups such as indole or pyrrole moieties [79]. The peak observed at 1112 cm⁻¹ corresponds to C–O stretching, while weaker bands below 700 cm⁻¹ may be related to substituted C–H groups in unsaturated systems [80]. Collectively, these spectral features are consistent with previously reported characteristics of fungal melanin, reflecting a complex polymer composed of aromatic and heterocyclic units [81–83]. The obtained FTIR profile closely resembles those described for melanins from various fungal sources [84–87].

Raman spectroscopy was further employed to investigate the structural characteristics of the purified pigment (Fig. 5C). The Raman spectrum exhibited a prominent band at approximately 1563 cm⁻¹, which is assigned to C=C stretching vibration within aromatic ring systems and reflects the indole-based polymeric backbone characteristic of eumelanin [88]. A second broad band centered at approximately 3427 cm⁻¹ was also observed and is attributed to O–H stretching vibrations arising from hydroxyl groups and hydrogen-bonded water molecules associated with the melanin polymer. The presence of this broad band reflects the hydrated and heterogeneous nature of naturally occurring melanins and has been reported previously for microbial melanin pigments. Collectively, the Raman spectral profile is consistent with previously reported eumelanin spectra and provides additional evidence supporting the identification of the extracted pigment as eumelanin [89, 90].

**Fig. 6:**
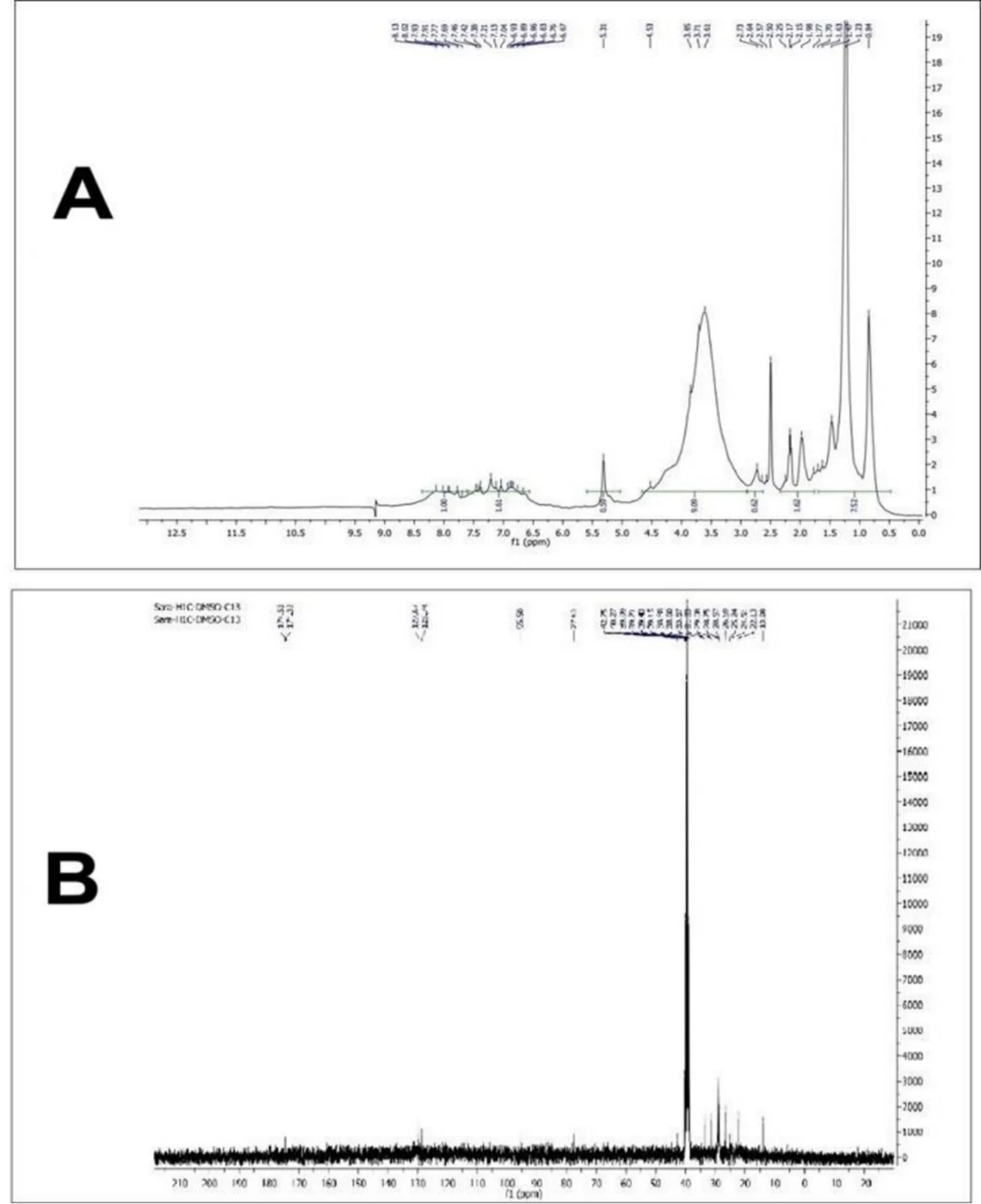
^1^HNMR (**A**); and ^13^CNMR (**B**) of extracted melanin produced by *A. terreus*

To further elucidate the structural features of the extracted melanin, both ¹H and ¹³C NMR analyses were performed (Fig. 6A, B). The spectra displayed signals in both aliphatic and aromatic regions, reflecting the complex and heterogeneous nature of the pigment. Analysis of the ¹H NMR spectrum revealed a broad resonance centered around ~ 8.0 ppm, which is indicative of hydroxyl functionalities associated with aromatic ring systems. The signals detected within the range of 6.5–7.3 ppm are characteristic of aromatic protons and are consistent with indole- and pyrrole-derived structural units commonly found in melanin polymers. Resonances appearing between 4.5 and 5.4 ppm may be attributed to vinylic protons (C=C–H) in proximity to heteroatoms such as nitrogen or oxygen [91]. A signal observed at approximately 3.3 ppm corresponds to residual water in DMSO-d₆, which overlaps with nearby peaks in the 3.2–4.2 ppm region. These signals are generally associated with methylene or methyl groups linked to heteroatoms (e.g., –CH₂–O), suggesting the presence of oxygenated side chains or partially oxidized aliphatic segments within the polymer structure [92]. In addition, signals within the 1.3–2.3 ppm range are likely related to aliphatic protons associated with nitrogen-containing functional groups. The broader aliphatic region (0.5–2.5 ppm) exhibited multiple overlapping peaks, which may arise from methylene bridges, lipid-derived residues, or other aliphatic components incorporated during pigment formation [93]. The ¹³C NMR spectrum further supported these findings, showing peaks around 40 ppm that may be associated with carbon atoms bonded to nitrogen (CH–N). Signals detected at 30, 24, and 15 ppm indicate the presence of aliphatic chains, while resonances at approximately 128–130 ppm correspond to aromatic carbon atoms, likely originating from indole-type structures. A distinct peak at 175 ppm suggests the presence of carbonyl groups within quinone-like structures, which are characteristic features of eumelanin quinones [94–96].

**Fig. 7:**
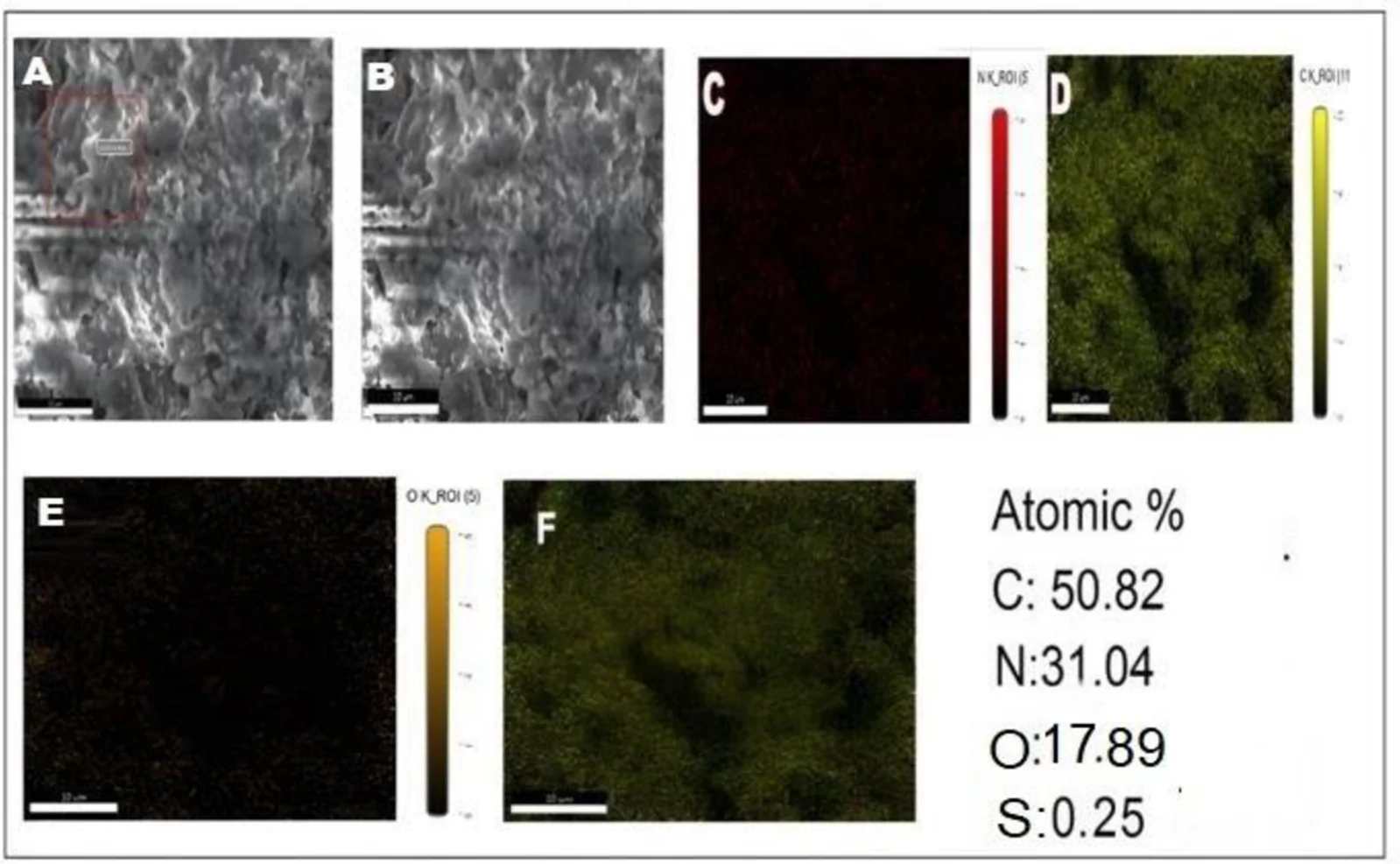
SEM images of extracted melanin produced by *A. terreus* at different magnifications (**A**, **B**); Energy dispersive X-ray spectroscopy (EDX) and elemental mapping analysis showing elemental composition of eumelanin (**C**–**F**)

Furthermore, this study conducted SEM, energy dispersive X-ray (EDX) spectroscopy and elemental mapping investigation of extracted melanin from *A. terreus* to confirm the relationship between the nitrogen content in this pigment and the absorption at 1391.13 cm^− 1^ (C−N stretching). This approach aids in identifying pigment structure and provides insights into its elemental composition [97, 98]. SEM images (Fig. 7A, B) revealed that the pigment formed irregular, amorphous aggregates, a morphology commonly associated with melanin. EDX analysis (Fig. 7C–F) indicated that the pigment was primarily composed of carbon (50.82%), nitrogen (31.04%), oxygen (17.58%), with low sulfur content (0.25%). The relatively high nitrogen content, together with the low sulfur percentage, provides important insight into the chemical nature of extracted eumelanin. Previous studies have shown that melanins synthesized via certain pathways contain minimal nitrogen, whereas nitrogen-rich melanins are associated with indolic structures [99–104]. In addition, the sulfur content can be used to distinguish between different melanin types, as pigments with very low sulfur levels are typically associated with eumelanin-like structures, whereas higher sulfur content is characteristic of alternative forms [71, 105–107]. These findings, together with the spectroscopic data (FTIR and NMR), are fully consistent with the structural characteristics strongly supports that the extracted melanin corresponds to a eumelanin-type biopolymer.

### Free radical scavenging activity

**Fig. 8:**
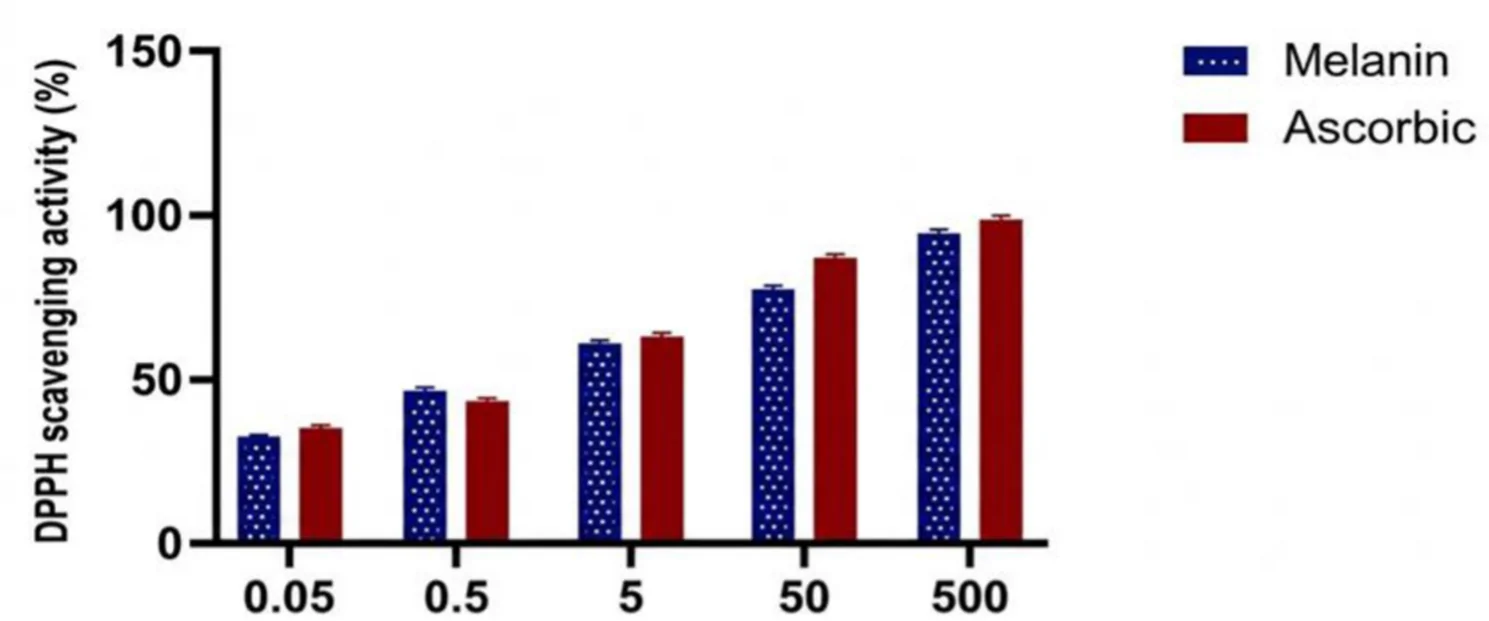
Antioxidant activity of eumelanin pigment produced by *A. terreus* AUMC1577 and ascorbic acid as standard

Oxidative stress is associated with several illnesses, such as cardiovascular disease, cancer, neurological disorders, and the aging process [108]. Hydroxyl radicals easily interact with biological molecules including proteins and DNA, which can cause health problems [109]. Finding safe and effective antioxidants that scavenge hydroxyl radicals is, thus, indispensable. In the present study, the extracted *A. terreus* eu-melanin pigment was tested for antioxidant activity against DPPH free radicals. The findings indicated that the DPPH scavenging activity of extracted eu-melanin pigment (30.5–96.8%) increased with increasing the concentrations (1.25–100 µg/mL), and showed a dose response relationship, with an average IC_50_ value of 17.1 µg/mL (Fig. 8). Extracted eu-melanin pigment has comparable antioxidant activity with standard ascorbic acid (IC_50_=16.83 µg/mL). The DPPH free radical scavenging activity of extracted eumelanin significantly exceeded that of melanin derived from *Streptomyces glaucescens* NEAE-H (57.2%) [110]. The existence of unpaired electrons in dopa-melanin’s chemical structures may explain its antioxidant efficacy. These electrons interact with oxidizing radicals via simple one-electron transfer mechanisms [111].

### In vitro cytotoxicity assay

**Fig. 9:**
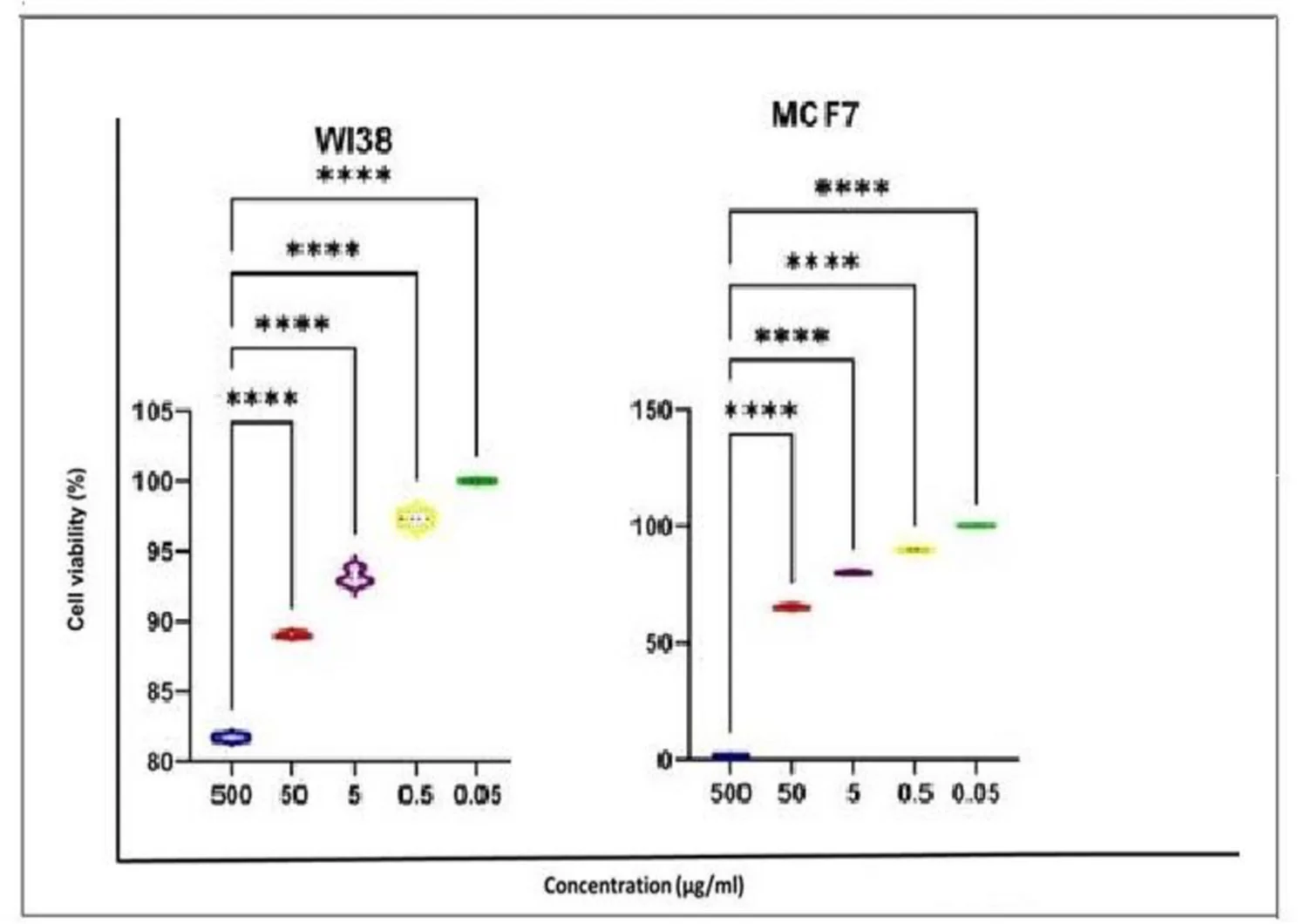
In vitro cytotoxicity and anticancer activities of various concentrations of the eumelanin pigment of *A. terreus*

Evaluation of cytotoxicity represents a critical step in the development of potential therapeutic agents [112]. In the present study, the cytotoxic activity of the extracted eumelanin was assessed using the MTT assay against a normal human lung fibroblast cell line (WI-38) and a breast cancer cell line (MCF-7). The results demonstrated that eumelanin exhibited negligible cytotoxicity toward the normal WI-38 cell line, with an IC₅₀ value exceeding 500 µg/mL, indicating favorable biocompatibility and preservation of normal cellular metabolic activity even at higher concentrations. In contrast, there was a noticeable decrease in cell viability of MCF-7 cells that was correlated with the dosage (Fig. 9).The calculated IC₅₀ value for MCF-7 cells was 68.77 µg/mL, reflecting a significant effect of eumelanin on breast cancer cells. These findings highlight a selective cytotoxic profile, where the eumelanin preferentially targets malignant cells while sparing normal cells. This selective behavior is consistent with previous reports on microbial melanins, which demonstrated minimal toxicity toward normal cell lines alongside inhibitory effects on cancer cells [113–115]. The cytotoxic activity of melanin may be associated with multiple mechanisms, including induction of apoptosis, modulation of cellular signaling pathways, and inhibition of tumor progression [116, 117]. Collectively, these findings support the potential of eumelanin as a new promising bioactive candidate for breast cancer-targeted applications.

### Molecular docking analysis and correlation with cytotoxic activity

Molecular docking was employed to investigate the potential interactions between eumelanin and key protein targets involved in breast cancer progression [118–119]. Two well-established targets were selected, namely breast cancer type 1 susceptibility protein (BRCA1) and estrogen receptor alpha (ERα), due to their central roles in tumor development and progression [120–122]. While BRCA1 is primarily associated with DNA repair and genomic stability, ERα is a major regulator of hormone-dependent breast cancer and is expressed in a large proportion of breast tumors [123–125]. Docking simulations (Table 5) demonstrated that eumelanin exhibited favorable binding interactions with both targets; however, a stronger affinity was observed toward ERα. The binding energy for ERα (− 8.0 kcal/mol) was notably lower than that observed for BRCA1 (− 6.6 kcal/mol), indicating a more stable ligand–receptor complex. At the molecular level, the interaction with BRCA1 involved multiple hydrogen bonds with key amino acid residues, including ARG1758, ARG1762, ASP1851, GLY1803, CYS1847, and GLN1848.

**Table 5:** Molecular docking report of eumelanin breast cancer protein interaction

Protein	Binding energy ΔG (kcal/mol)	3D interaction	2D interaction
Estrogen Receptor Alpha	−8.0		
Breast Cancer Type1 Susceptibility Protein	−6.6		

In contrast, the interaction with ERα was characterized by a more stable docking conformation, supported by hydrogen bonding with residues such as LYS531, ASP351, ASN519, and GLU523, in addition to multiple hydrophobic interactions within the binding pocket. The coexistence of polar and nonpolar interactions suggests a complex binding mode that enhances ligand stability within the active site [126]. The observed binding behavior can be attributed to the structural features of the eumelanin, which is composed of aromatic and heterocyclic units enriched with functional groups capable of participating in hydrogen bonding and electrostatic interactions. Such structural characteristics are known to facilitate strong interactions with protein targets [127]. These computational findings provide preliminary insights into the potential molecular interactions of eumelanin with breast cancer-associated proteins.

The molecular docking results were consistent with the in vitro cytotoxicity findings obtained in MCF-7 breast cancer cells. The reduction in MCF-7 cell viability observed in the MTT assay, together with the minimal cytotoxicity toward normal WI-38 cells, may be associated with the predicted interaction between eumelanin and ERα, a key driver of breast cancer cell proliferation. These observations support the hypothesis that ERα may represent a relevant molecular target for eumelanin; however, this proposed mechanism requires experimental validation. Collectively, the integration of the docking and cytotoxicity findings identifies ERα as a potential molecular target worthy of further investigation and supports future mechanistic studies on the interaction between eumelanin and hormone-responsive breast cancer cells.

The present study has several limitations. The cytotoxicity assessment was based on the MTT assay using a single breast cancer cell line and therefore reflects changes in cellular metabolic activity rather than confirming apoptosis, antiproliferative mechanisms, or therapeutic selectivity. In addition, the molecular docking results provide predictive evidence only and require validation through receptor-based assays, mechanistic studies, and in vivo models. Further investigations using additional breast cancer subtypes, normal mammary cells, and animal models are therefore warranted.

## Conclusion

This study reported the isolation of a melanin-producing *A. terreus* strain from the Wadi Allaqi Biosphere Reserve and demonstrates its potential as an efficient microbial cell factory. Statistical optimization significantly enhanced pigment yield, while comprehensive physicochemical and spectroscopic analyses confirmed its structural characteristics consistent with eumelanin. The extracted eumelanin exhibited remarkable antioxidant activity and selective cytotoxic activity against MCF-7 breast cancer cells with minimal effects on normal WI-38 cells. Molecular docking further supported these findings, revealing a strong interaction with ERα, suggesting a potential mechanism underlying its cytotoxic activity. Overall, this work highlighted the biotechnological and biomedical potential of microbial eumelanin and provided new insights into its application in breast cancer-targeted studies; however, additional mechanistic studies and in vivo investigations are required to further validate its therapeutic applicability.

## Supplementary Information

Below is the link to the electronic supplementary material.

Supplementary Material 1.

## Data Availability

No datasets were generated or analysed during the current study.

## Footnotes

1 SS, Sum of Squares; DF, Degrees of Freedom; MS, Mean Square; CV, Coefficient of Variation; Values marked with an asterisk (∗) indicate terms that are statistically significant (*Ρ*<0.05).
