# Supplementary material for: Microbial production of eumelanin from a desert-derived Aspergillus terreus: statistical optimization, comprehensive characterization, and in silico insights into breast cancer-associated targets
Source: Microb Cell Fact. 2026 Sep 26;25:190. doi: 10.1186/s12934-026-03114-7 (PMC13615586; doi:10.1186/s12934-026-03114-7)
Supplement: Supplementary file 1 [file 12934_2026_3114_MOESM1_ESM.docx]

Fig S1. Visual observation of melanin production by *A. terreus* (A) At 0 time before incubation,(B) Color change after 7 days incubation.

Fig S2. TLC analysis of extracted melanin pigments produced by *A. terreus* (B) compared with standard synthetic melanin (A).

**Table S1 . Experimental independent variables used for the production of melanin by F11 using Plackett Burman design.**

| # | Variables | Unit | Symbol | Experimental value | |
| --- | --- | --- | --- | --- | --- |
|  |  |  |  | Low (- 1) | High (+1) |
| 1 | pH |  | A | 5 | 9 |
| 2 | Temperature | ^0^C | B | 25 | 35 |
| 3 | Inoculum volume | CFU/mL | C | 1 | 3 |
| 4 | Incubation period | Days | D | 7 | 14 |
| 5 | L-tyrosine | g/L | E | 3 | 7 |
| 6 | Potato | g/L | F | 150 | 250 |
| 7 | Ferrous sulphate | g/L | G | 0.1 | 1 |
| 8 | Sodium dihydrogen  phosphate | g/L | H | 10 | 14 |
| 9 | Dextrose | g/L | J | 15 | 25 |
| 10 | Copper sulphate | g/L | K | 0.1 | 1 |
| 11 | Calcium chloride | g/L | L | 0.5 | 1 |

**Table S2. Experimental variables for Box-Behnken design at different levels for F11**

| Factors | Variables | Units | Experimental values | | |
| --- | --- | --- | --- | --- | --- |
|  |  |  | Low  (- 1) | Intermediate  (0) | High (+1) |
| A | Inoculum volume | CF U/ mL | 1 | 2 | 3 |
| B | L-Tyrosine | g/L | 3 | 5 | 7 |
| C | Sodium dihydrogen phosphate | g/L | 10 | 12 | 14 |

**Table S3. Physicochemical properties of melanin produced by *A. terreus* compared with standard synthetic melanin**

| Property | Treatment | Results | |
| --- | --- | --- | --- |
|  |  | Purified melanin of *A. terreus* | Synthetic melanin |
| **Color** | **Observation** | dark brown | Black |
| **Solubility** | Distilled water | Soluble | Soluble |
|  | Methanol | Partially soluble | Soluble |
|  | Absolute ethanol | Partially soluble | Soluble |
|  | Chloroform | Partially soluble | Soluble |
|  | Benzene | Partially soluble | Soluble |
|  | Acetone | Partially soluble | Soluble |
|  | Ethyl acetate | Partially soluble | Soluble |
|  | 1Mol/L KOH | Soluble | Soluble |
|  | 1Mol/L NaOH | Soluble | Soluble |
|  | DMSO | Soluble | Soluble |
| **Stability** | Heat (60° C for  2, 4, 6 h, respectively) | Stable | Stable |
|  | Heat (80° C for  2, 4, 6 h, respectively) | Stable | Stable |
|  | Heat (100° C for 2, 4, 6 h, respectively) | Stable | Stable |
|  | Sun light (treatment for10 days) | Stable | Stable |
| **Precipitation with** | 3N HCl | Precipitated | Precipitated |
|  | 1% FeCl3 | Precipitated | Precipitated |
| **Oxidation** | Hydrogen peroxide | Decolorization (oxidized) | Decolorization (oxidized) |
| **Bleach test** | KMNO_4_ | Decolorization | Decolorization |
